# Balanced-force shim system for correcting magnetic-field inhomogeneities in the heart due to implanted cardioverter defibrillators

**DOI:** 10.3389/fmed.2024.1225848

**Published:** 2024-02-13

**Authors:** Mirko Hrovat, Aravindan Kolandaivelu, Yifan Wang, Anthony Gunderman, Henry R. Halperin, Yue Chen, Ehud J. Schmidt

**Affiliations:** ^1^Mirtech, Boston, MA, United States; ^2^Medicine (Cardiology), Johns Hopkins University, Baltimore, MD, United States; ^3^Georgia Institute of Technology, Atlanta, GA, United States

**Keywords:** ICD, defibrillator, implant, MRI, metal artifacts, shimming, susceptibility, artifact reduction

## Abstract

**Background:**

In the US, 1.4 million people have implanted ICDs for reducing the risk of sudden death due to ventricular arrhythmias. Cardiac MRI (cMR) is of particular interest in the ICD patient population as cMR is the optimal imaging modality for distinguishing cardiac conditions that predispose to sudden death, and it is the best method to plan and guide therapy. However, all ICDs contain a ferromagnetic transformer which imposes a large inhomogeneous magnetic field in sections of the heart, creating large image voids that can mask important pathology. A shim system was devised to resolve these ICD issues. A shim coil system (CSS) that corrects ICD artifacts over a user-selected Region-of-Interest (ROI), was constructed and validated.

**Methods:**

A shim coil was constructed that can project a large magnetic field for distances of ~15 cm. The shim-coil can be positioned safely anywhere within the scanner bore. The CSS includes a cantilevered beam to hold the shim coil. Remotely controlled MR-conditional motors allow 2 mm-accuracy three-dimensional shim-coil position. The shim coil is located above the subjects and the imaging surface-coils. Interaction of the shim coil with the scanner’s gradients was eliminated with an amplifier that is in a constant current mode. Coupling with the scanners’ radio-frequency (rf) coils, was reduced with shielding, low-pass filters, and cable shield traps. Software, which utilizes magnetic field (B_0_) mapping of the ICD inhomogeneity, computes the optimal location for the shim coil and its corrective current. ECG gated single- and multiple-cardiac-phase 2D GRE and SSFP sequences, as well as 3D ECG-gated respiratory-navigated IR-GRE (LGE) sequences were tested in phantoms and *N* = 3 swine with overlaid ICDs.

**Results:**

With all cMR sequences, the system reduced artifacts from >100 ppm to <25 ppm inhomogeneity, which permitted imaging of the entire left ventricle in swine with ICD-related voids. Continuously acquired Gradient recalled echo or Steady State Free Precession images were used to interactively adjust the shim current and coil location.

**Conclusion:**

The shim system reduced large field inhomogeneities due to implanted ICDs and corrected most ICD-related image distortions. Externally-controlled motorized translation of the shim coil simplified its utilization, supporting an efficient cardiac MRI workflow.

## Introduction

The implantable cardioverter-defibrillator (ICD) case is a small [5x5x1cm^3^] battery-powered box implanted over the rib cage, but under the skin. The ICD’s case has one or two attached electrical cables that lead from the box into chambers of the heart. These cables are used to detect irregular electrical signals (arrythmia), analyze their nature, and thereafter deliver strong shocks that stop lethal forms of rapid heartbeats. ICDs are typically implanted in patients with weak hearts, often in the setting of coronary artery disease, and other conditions that increase risk of death from a rapid heart rhythm, termed sustained Ventricular Tachycardia (VT) and Ventricular Fibrillation (VF). The ICD continuously monitors the heartbeat and delivers electric shocks, when needed, restoring a normal heart rhythm.

Approximately 1.4 million people in the US (2021) (1, 2) have implanted ICDs and ~ 50% of these require an MRI scan over the course of their lifetime (3, 4). While most patients with implanted ICDs can have diagnostic quality MRI studies in anatomical regions distant from the heart (4, 5), a major portion of the population that require heart studies can have severely lower quality imaging studies (5–7). The ferromagnetic transformer present in every ICD perturbs the magnetic field uniformity in the heart to a larger degree than today’s cardiac MR (cMR) sequences can contend with, resulting in the appearance of image voids which render heart images partially unreadable.

The cMR technique of most interest for VT and VF management is Late Gadolinium Enhancement (LGE), a respiratory and cardiac-gated Inversion-Recovery 2D or 3D sequence. LGE is the optimal means to visualize scar tissue in the heart, the main cause of VT arrhythmia, a disease that causes sudden cardiac arrest in 0.35 M patients yearly (US, 2020) (8). Mapping the scar location with LGE is a key step in treating this disease with catheter ablation, since this information is used for procedure planning at electrophysiology (EP) clinics. Today, a large fraction of patients with ICDs are referred to CT, NM or PET instead of MRI (9) but these provide inferior resolution of the scar’s 3D geometry and composition.

LGE cMR is of particular interest for planning ablation treatment in patients with VT, most of whom have an implanted ICD present at the time of imaging. The large number of patients with implanted ICD’s is a result of the success of ICDs in preventing sudden death due to VT and VF (10). However, after ICD implantation, VF and VT episodes can lead to ICD shocks which are unpleasant and reduce cardiac output. When these shocks occur despite pharmaceutical treatment to reduce the arrythmia episodes, patients are referred to catheter ablation. A pre-requisite step to ablation is identification of the myocardial scar regions that cause arrhythmia. MRI is used for such interventional procedure planning, with MR Angiography (MRA) providing the anatomy of the cardiac chambers (11, 12) and LGE used to locate the scar in the ventricular walls (13–15). This data is then sent to the electro-anatomical (EAM) workstation used during the EP interventional procedure so it can be used to guide catheter navigation to the ablation target(s). Additionally, MR imaging of patient groups with ICDs can be helpful in guiding the need for immunosuppressive medical therapy or coronary revascularization (15).

**Figure 1:**
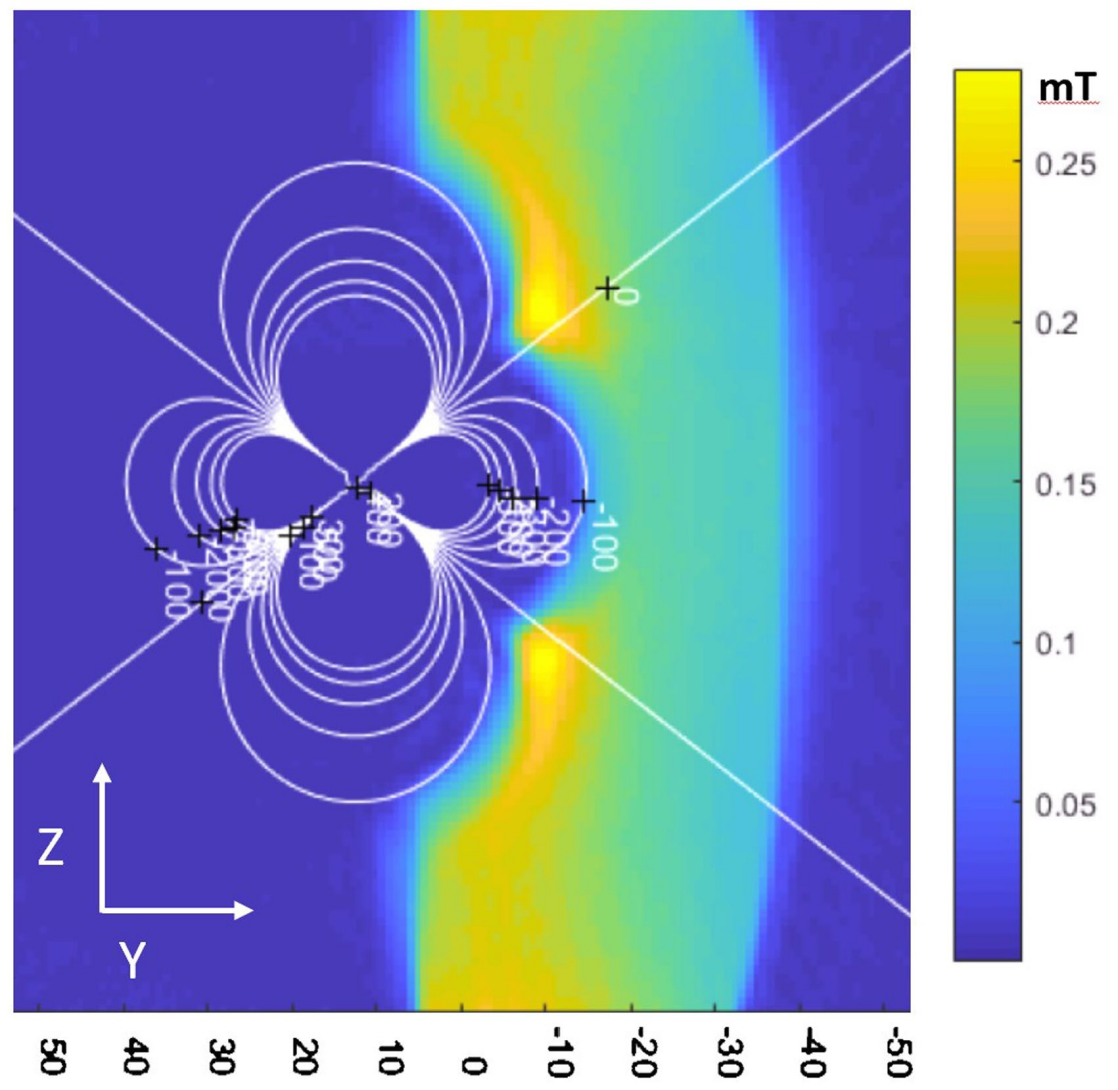
Typical ICD-related dipolar magnetic field shown on a sagittal magnetic field map obtained with an ICD placed on a cylindrical phantom. Contour steps are 100 ppm increments.

Imaging the left-ventricular (LV) wall scar tissue is difficult in a large fraction of patients with implanted ICDs. In a review of pre-VT ablation studies done at Johns Hopkins since 2001, 29% of 2D LGE and nearly all 3D LGE studies had limited interpretation due to the presence of an ICD (15). The ICD shock is delivered by amplifying the voltage from a battery, which requires use of a ferromagnetic transformer. When placed in the MRI scanner’s high magnetic field, this ferromagnetic transformer becomes magnetized, creating a strong magnetic field of its own whose shape is described by a simple magnetic dipole (Figure 1) oriented along the scanner static (B_0_) magnetic field direction (z). As seen, the z-component of the dipole field consists of three lobes, with the central toroidal lobe penetrating 10–20 cm into the subject’s chest. Since ICDs are implanted in the left chest wall, above the rib cage, this dipole-shaped magnetic field reaches parts of the heart, such as the LV wall, creating a highly inhomogeneous field of >100 Parts Per Million (ppm). This local inhomogeneous field exceeds the compensatory range of the MRI scanner’s integral shim coils, which can correct only much smaller fields (~20 ppm). Since the frequency bandwidth of an MRI sequence’s radio-frequency (rf) pulses (typically <2Khz at @1.5 T) is also less than the inhomogeneity bandwidth (~6Khz @1.5 T), the uncorrected field distortion is not excited by the pulse, and therefore typically appears as a dark hole in the image, surrounded by additional regions of distorted shape.

**Figure 2:**
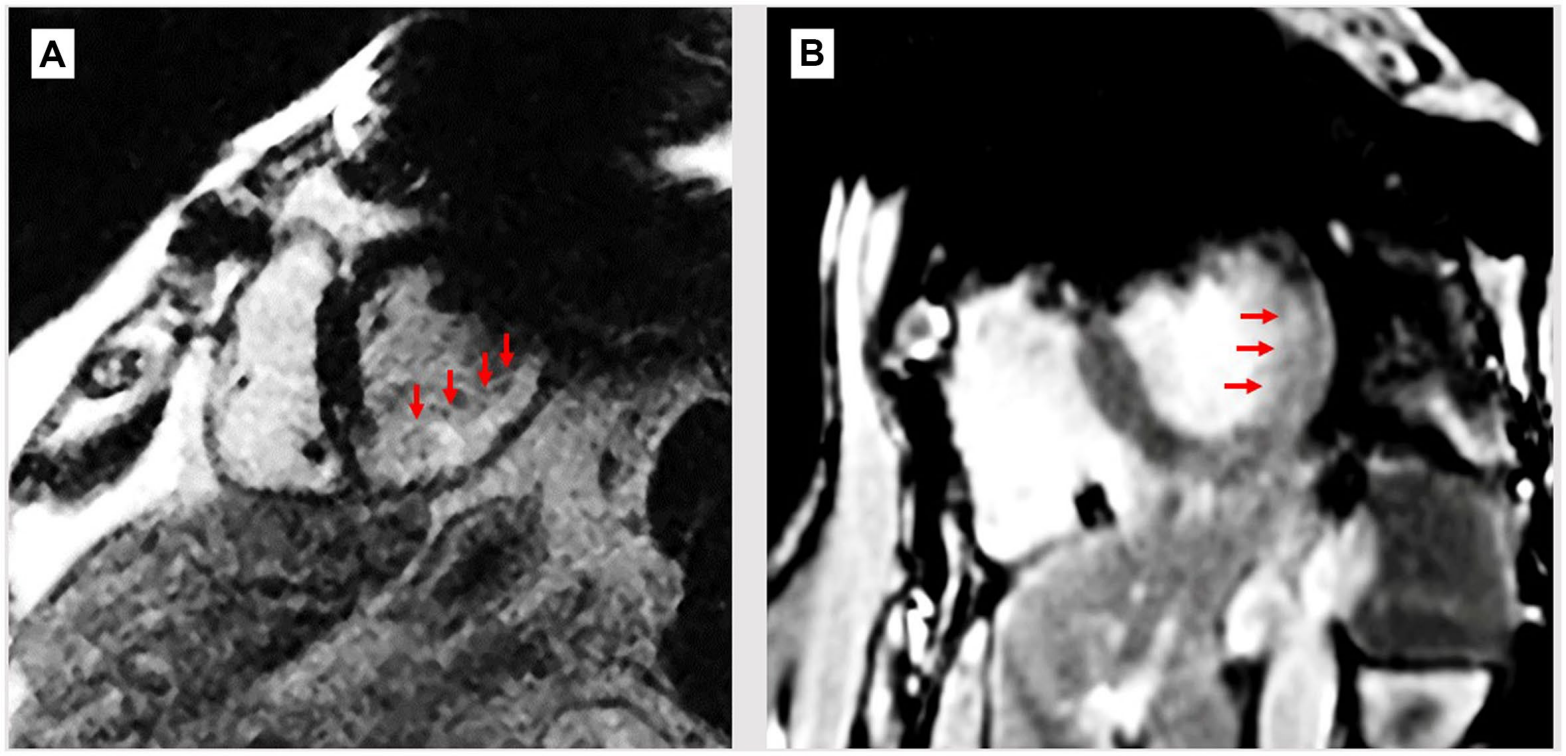
Breath-held wideband LGE scans of patients with ICDs and myocardial infarcts. **(A)** ICD-generated void still covers upper left of LV wall, possibly hiding extension of scar (bright region, red arrows). **(B)** Patient with thin mid-wall scar (arrows) which may extend into the void region, which remains despite WB sequence.

Wideband (WB) MRI sequences were developed to correct artifacts of <25 ppm, and they do reduce the ICD artifact volume (e.g., the exterior of the affected area), but they are unable to entirely correct it (14). Figure 2 shows WB-LGE images of two patients, post-infarction with LV scars, where there may be extensions of the scar into the residual regions. Orthopedic-implant-focused methods (MAVRIC, SEMAC) (16), used for removing large susceptibility artifacts from metallic orthopedic implants are not applicable for the heart due to the long scan times they would require in physiologically moving anatomy, where lack of synchronization is also difficult to correct for because cardiac motion results in substantial tissue deformation. Implanting the ICD case in the right chest wall can reduce the magnetic field inhomogeneity over the heart (15). However, because the ICD case serves as one of the two current terminals of the defibrillator, standard left sided positioning is favored for an effective shock treatment (17).

Addressing the ICD generated magnetic field inhomogeneity problem requires placement of dedicated resistive shim coils inside the bore. Shim coils placed within the scanner must not displace during stretcher movement, or during time-varying gradient-currents, so the forces and torques on these coils must be balanced. Furthermore, the shim coil must not couple with the rf coils and gradient coils already present in the MRI scanner. Most existing shim-coil prototypes (18, 19) are intended for head applications, while the few existing full-body systems can only correct minor (~7 ppm) abdominal inhomogeneities that occur at tissue-air boundaries in patients without implanted ICDs (20, 21).

The project goal was to develop a shim-coil that could deliver the large magnetic field corrections required to render ICD-corrupted cMR images useful for diagnostic imaging by lowering the residual inhomogeneity in a user selected Region of Interest (ROI) to below 25 ppm. Since the coil is present within the MRI bore, it should not move due to the presence of the large static magnetic field (B_0_) gradients present when the coil is inserted or withdrawn from the bore (e.g., at the border of the gantry). Furthermore, as any coil inserted into the MRI bore interacts with the existing hardware in the bore, such as the gradient and rf coils, it is necessary to validate that the coil would not reduce the MRI’s signal-to-noise ratio, or lead to image distortion.

Additionally, correcting the ICD-generated magnetic inhomogeneity should not lengthen conventional cMR procedures exorbitantly. Finally, we desired to make the field correction procedure performance relatively easy for the usual MRI techs, not requiring a larger staff, or forcing multiple entries into the MRI room. To tackle these issues, we developed programs to compute the optimal three-dimensional location of the shim coil and the current strength required for compensation, as well as a remotely-controlled robotic system that cantilevered the shim coil inside the bore, and permitted accurate displacement of the shim coil to the desired location by commands delivered from outside the room. Lastly, we designed and tested methods to iterate around the (theoretical) settings provided by the shim optimization programs, utilizing fast imaging sequences to fine-tune the settings and observe the benefits in seconds.

## Methods

### Cardiac shim system requirements

The CSS needed to meet MRI environment safety requirements; (i) the system could not displace or rotate by action of the static magnetic field (B_0_), the gradient magnetic field or the interaction between those fields and the current in the shim coil (which creates a magnetic field of its own). (ii) Use of the CSS within the bore could not cause additional subject rf heating during MR imaging, relative to the IEC/FDA heating limits (1.5°C/Kg) permitted during human imaging in the scanner. The CSS outer frame also had to be completely insulated from DC or AC electric currents. The system was constructed to meet MR Imaging requirements; its presence and use could not negatively affect the MR Image (iv) Contrast to Noise Ratio or (v) spatial linearity, as measured relative to the scanner performance without the shim system in-place. (vi) Use of commercial MRI surface coils had to be supported.

The CSS performance was specified to (vii) reduce ICD- generated field inhomogeneity on a user-defined 5x5x5 cm^3^ ROI in the heart from 100 ppm to 25 ppm at all locations, with the ICD-related void locations <10 cm posterior to the patient’s abdominal surface. (viii) The imaging improvement should be achieved rapidly, to minimally elongate the duration of the MRI protocol, with the patient typically breath-holding or under General Anesthesia over this time, and (ix) require minimal human interaction within the MRI room during the procedure.

### Shim coil for strong directional magnetic field induction

**Figure 3:**
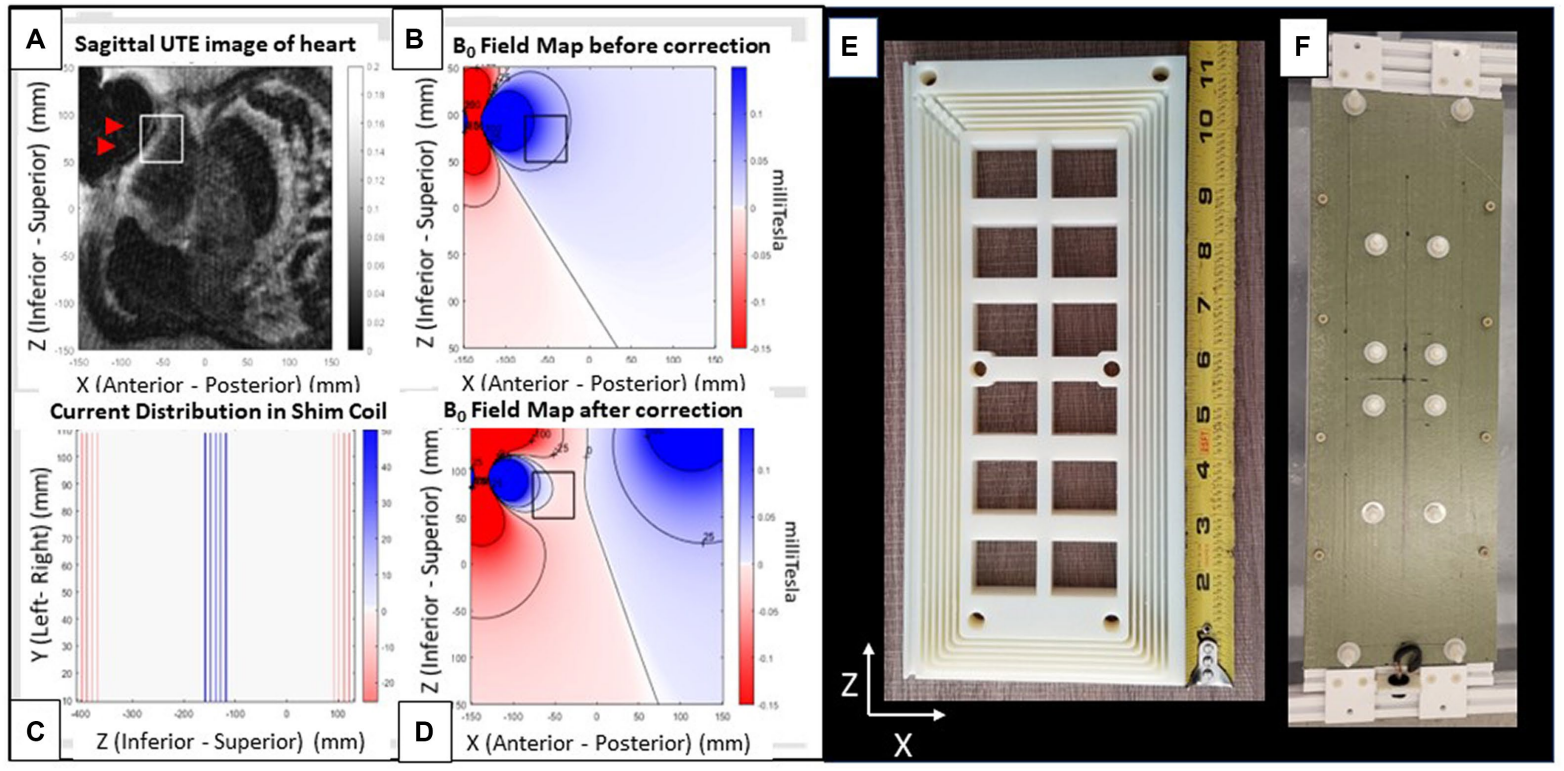
Shim coil. (A–D) Images from a patient with an implanted ICD. **(A)** Radial UTE MR image with target ROI region (white square) and void (black) region (arrows) indicating region of largest ICD generated field inhomogeneity. **(B)** Simulated field map of a dipole fitted to the UTE field map, with dark colors indicating regions of largest inhomogeneity (Blue: positive (higher) field, red: Negative (lower) field. Field contours lines (black) are at 25 ppm increments). **(C)** 2D coronal current pattern of the shim coil, with color indicating current polarity. **(D)** Simulated total field after shim-coil correction. Note far smaller changes within the box. Views of shim coil **(E)** interior form of one out of the two coils composing the complete shim coil and **(F)** fully assembled exterior. The rectangular coil is oriented with its long axis along the scanner’s superior–inferior (z) and its short axis along the left–right (x) directions. It produces a magnetic field along the anterior–posterior (y) direction.

The shim coil design was focused on minimizing the inhomogeneity within a user-specified target ROI (white and black squares in Figures 3A,B,D). Based upon field maps obtained in humans with ICD’s and with ICD’s placed on phantoms, it was determined that the inhomogeneity created by ICD was well described by a dipole. The coil winding pattern was obtained by minimizing the inhomogeneity in the target ROI, based upon current segments. It was observed that the optimal current segment configuration could be well approximated by a single shim coil.

The total magnetic force on the coil was balanced by return currents that were well separated from the central segments (Figure 3C), effectively creating two coil halves. The B_0_ field creates a torque on the two coil halves to fold the coil which is easily compensated by a fiberglass sheet stiffener. To reduce the maximum required current (~10A) to meet the shimming requirements (75 ppm), 72 coil turns in the central segment were used. The shim coil (Figure 3) was designed to provide a magnetic field of 0.018 mT/Amp-current at a 15 cm distance from the coil surface. This large current per Amp current should require less than 10Amperes of direct-(electrical)-current (DC) to correct ICD artifacts deep below the anterior skin to the prescribed extent (75 ppm) over the specified ROI (5x5x5cm^3^).

To localize the shim coil location within the bore, a T-shaped fiducial marker was placed 2 cm below the center of the coil. This fiducial marker was filled with Gd-DTPA-doped water, so it could be easily observed with all MRI imaging sequences and used to register the shim coil location in the MRI-scanner coordinate frame. Once this registration was completed, the shim coil could be brought to any desired location using its supporting cantilevered arm.

### Penetration-panel electronics prevent RF interference from outside the MRI room

Power to the shim coil is provided by an amplifier in current controlled mode that is located outside the MRI room. To prevent RF noise (RFI) from entering the MRI room, a custom-built multi-stage low-pass RF filter was designed and built to support 15 Ampere currents and placed at the room’s penetration panel. The custom filter design is necessary to insure stable operation with the current controlled amplifier.

### Power amplifier ensures stable current supply to shim coil during MRI imaging

Power to the shim coil was provided by an AE Techron 7224 or 7,234 amplifier. The current control feedback loop insures correction of the output DC current even when it detects induced currents in the shim coil from the MRI scanner’s gradient coils, maintaining the net output magnetic field of the shim coil at the prescribed levels.

### Remotely activated cantilevered shim-coil displacement system to move the shim coil within the bore

**Figure 4:**
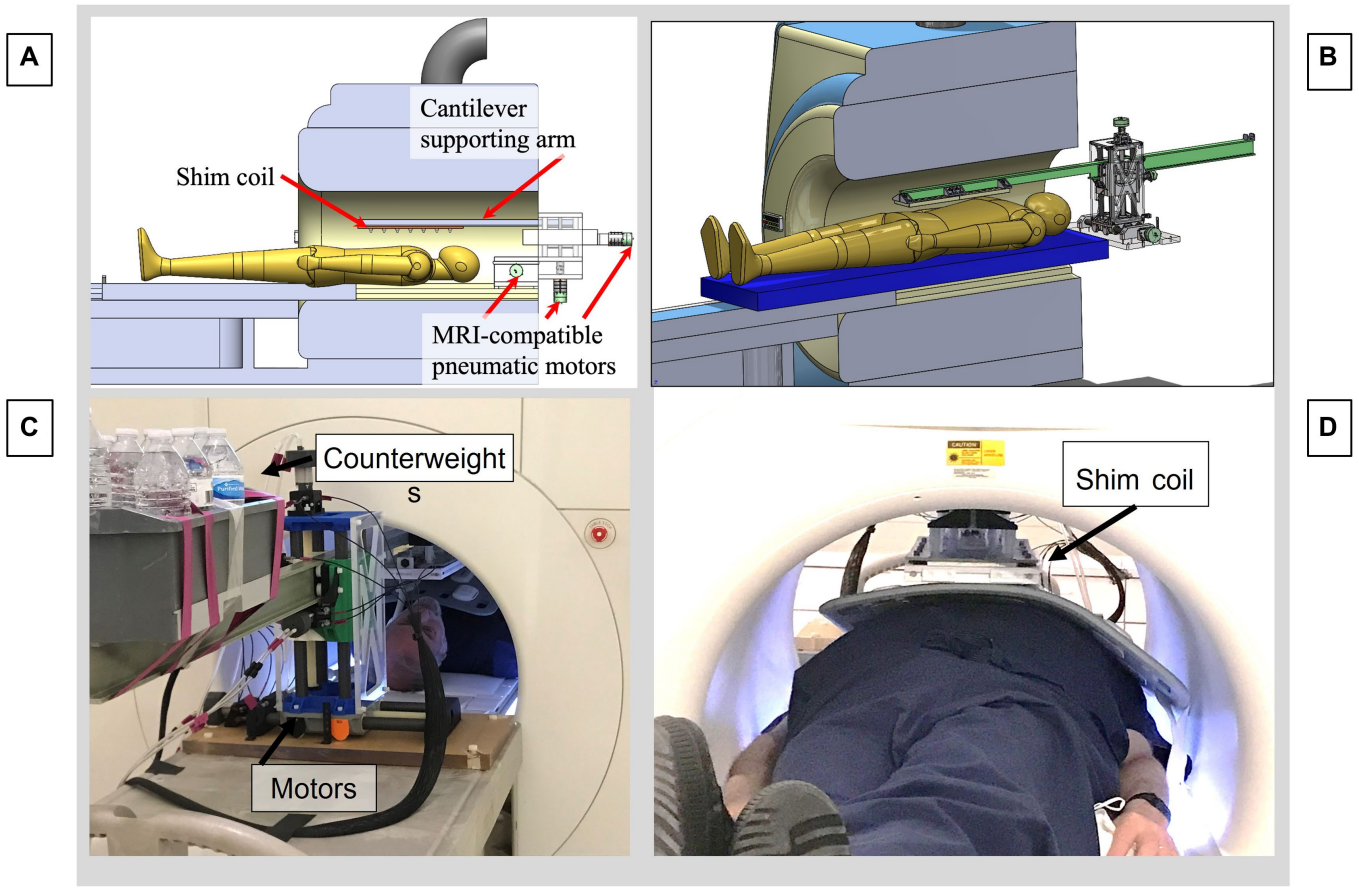
Motorized cantilevered system to remotely position the shim coil in the MRI. **(A)** CAD model of the entire system. **(B)** CAD focusing on cantilevered arm and motors. **(C)** View from rear of MRI bore, showing pneumatic motors and back end of cantilevered beam, with counterweights balancing the moment arm produced by the shim coil. **(D)** View from front of bore, showing subject inside the bore with a cardiac coil above the abdomen with the shim coil above, suspended from the distal end of the beam.

The shim coil is attached to a remotely activated 3-stage cantilever platform that enables movements along the scanner X (patient right–left), Y (patient anterior–posterior) and Z (patient superior–inferior) directions in the MRI bore. As shown in Figure 4, the platform that supports the cantilevered arm is situated immediately outside the scanner bore, with the cantilevered beam protruding up to 180 cm into the bore. The shim coil is suspended from the beam at its distal end. The allowable motion is 140 mm in the X, Y, and Z directions at a 2 mm precision, and the beam supports a 5 kg shim coil. A volume of water was added to the back end of the cantilevered arm, serving as counterweight, to balance the torque created by the shim coil, which is suspended from the front end of the beam. Linear translation of the beam in 3 directions was provided by lead screws, which were driven by pneumatically actuated motors, each equipped with an optic quadrature encoder for rotation angle feedback and closed-loop control (12). The optical fibers for the encoders and the air hoses driving the motors, were connected to a control box located outside the MR room via a waveguide, allowing for remote control of the cantilevered arm. The control box was connected to a PC-based (Matlab) program with a user interface designed to enter the desired target position of the shim coil, to the control box, and allowing the user to initiate motion. To ensure MR-compatibility, the components of the platform were either 3D-printed using PLA and resin, or made of acrylic, nylon, fiberglass, and other plastic materials.

### B_0_ field mapping and optimization of shim coil location and current

Two approaches are possible for optimization of the shim coil location and current. The first approach is deterministic and uses field maps, dipole fitting, and software optimization of shim coil location and current. The second approach is interactive and uses real-time imaging to optimize the shim coil location and current.

For the deterministic approach, static (B_0_) Magnetic field maps are acquired using double-echo MRI sequences, with the unwrapped phase-difference between the 1st and 2nd echo (TE_1_, TE_2_) phase-maps, forming the basis for the B_0_ field map. They were obtained with conventional cartesian gradient-echo, with a stack of radials self-navigated scan, as well as with ultrashort echo time (UTE) stack of spirals scans. The latter two imaging sequences had a shorter TE, leading to less signal dephasing, as well as a larger frequency bandwidth (>6KHz), which allowed obtaining correct B_0_ field maps closer to the largest magnetic field inhomogeneities within the ICD-caused perturbation volume, since imaging resulted in volumetrically smaller image voids. The cartesian GRE images were acquired with TE_1_ = 1,490 μs and TE_2_ = 1740 μs. The UTE images were acquired with TE_1_ = 50 μs and TE_2_ = 300 μs.

After computing the B_0_ Field map, the dipole strength and location is determined. This is done by fitting the dipole field profile to the field map data using Matlab code using a reduced number of points from the field map in the vicinity of the distortion. It is necessary to perform a coarse grid search before fine tuning the final fitting process. One advantage of the dipole model is that it allows a determination of the field profile in those regions where there is no MR signal. An example of this is shown in Figure 3B. To determine the position and current strength of the shim coil, a second step involves locating an ROI on the dipole field map and then optimally fitting the strength and position of the shim coil to reduce the inhomogeneity in the ROI. This process is illustrated with Figure 3D where the ROI is indicated as a black square and by comparison with Figure 3B the field is reduced nearly to zero inside the ROI. The calculations were performed with code written in Matlab.

**Figure 5:**
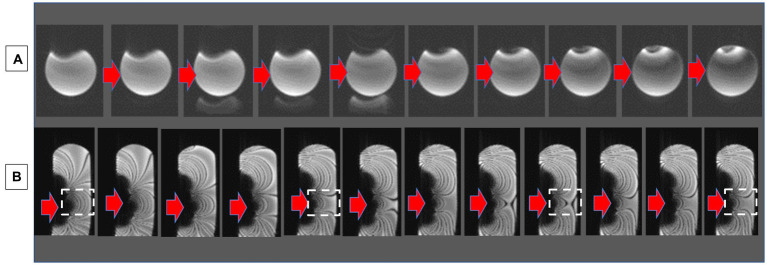
Interactive shimming in a phantom with an overlaid ICD, which causes an image void. **(A)** Changing the shim current with continuous axial GRE visualization, leading to a smaller void. **(B)** Changing the shim current with continuous sagittal SSFP visualization. Note the increasingly larger distances between the SSFP bands. The white square indicates the region corrected. Red arrows indicate the direction of progression for the current changes.

Alternatively, an interactive approach is also possible. Any real-time imaging sequence of a prescribed slice may be set up and observed while the current and position of the shim coil is adjusted during the scanning. This is shown in Figure 5. This approach could also be combined with the deterministic approach where the latter approach determines the initial parameters for the shim coil and the interactive approach is used to fine tune the result.

### Imaging protocols (sequences, phantoms, animal models) used to validate the system

We employed 2D and 3D Gradient- Recalled Echo (GRE) sequences, without and with preceding inversion-recovery (IR) pulses, as well as 2D Steady State Free Precession (SSFP) sequences, to validate system performance. ECG-gated single- and multiple- cardiac-phase sequences were used, as well as IR-GRE sequences (LGE) to emulate patient scanning conditions.

Following tests in phantoms, anesthetized swine (*N* = 3, weight 25-35Kg) were studied, with customary breath-held or respiratory-navigated ECG-gated sequences employed to reduce physiological motion artifacts. The ICD artifacts observed in patients with implanted ICDs were emulated experimentally by placing ICD cases on top of MRI phantoms, and on top of the rib-cages of the swine models.

**Figure 6:**
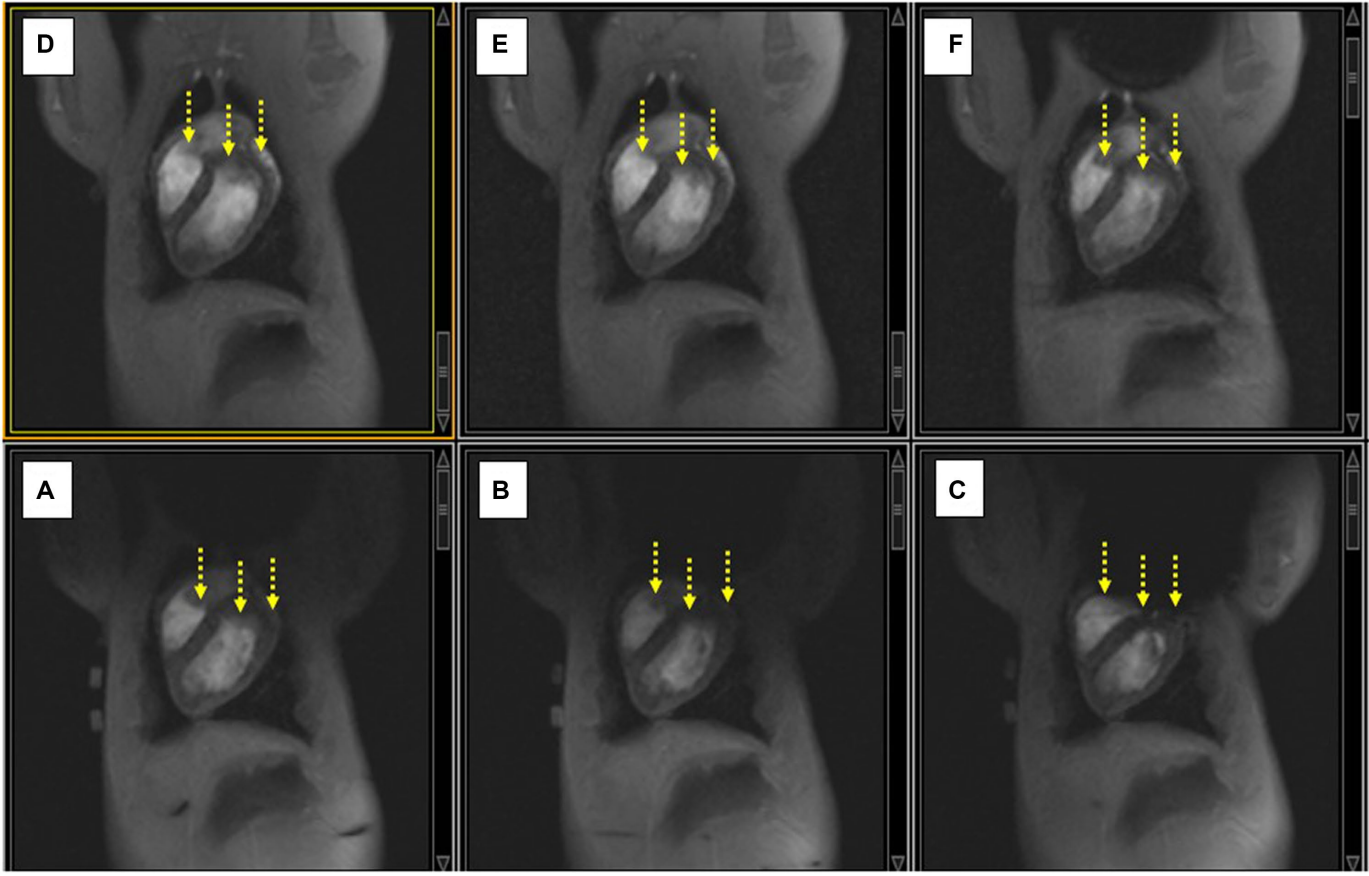
**(A–C)** Displacing the ICD case increasingly lower along the SI direction on the swine chest caused an increase in the extent of the non-uniformity artifact (black void), and additional signal-loss changes (yellow arrows). **(D–F)** Applying shim current restores the observed LV in each case compared to the image below.

In the swine (Figure 6), the ICD locations were carefully chosen to mirror the location of ICD artifacts in patients, which primarily consist of artifacts at the LV base (e. g. the superior portion of the LV) and on the anterior (i.e., abdominal) portions of the LV wall. Since the chest diameter of young (<0.5 years old) swine is equivalent to that of “skinny” human subjects, the artifacts in swine should mirror those seen in skinny patients, and overestimate those observed in more obese patients.

Medium size American College of Radiology (ACR) resolution phantoms (22) were employed to validate that the insertion of the CSS assembly into the MRI bore, along with execution of MRI sequences with varying parameters, did not reduce the spatial resolution or introduce geometric distortions, relative to scanner performance without the shim coil (i.e., with it outside the bore).

Since the MRI gradients induced currents into the shim coil during the imaging sequence’s gradient ramp and falls, MRI imaging was always performed with the CSS power amplifier (AE Techron 7224 or 7234, Elkhard, IN, USA), so that the, so that the amplifier’s feedback loop could balance out any induced currents. To test for image distortion, two scans with a given sequence were run at the same slice locations, but with the phase-encoding and frequency encoding directions interchanged between successive scans. The resulting images were subtracted to provide a measure of the distortion.

### Correcting B_0_ inhomogeneity

Optimal artifact reduction requires performing two settings accurately: correctly locating the shim coil and driving the correct current through the shim coil. The combination of both creates the correct magnetic field at the desired inhomogeneity location. Gross corrections were performed by bringing the shim coil to the software prescribed location and driving the recommended current (magnitude and direction) from the power amplifier. Iterative correction of the shim-coil location or current was performed by setting the MRI scanner for continuous (1–2 frames-per-minute) imaging of ECG-gated 2D GRE or 2D SSFP images, while moving the shim coil location in fixed increments, or changing the shim current magnitude in fixed increments.

Balanced SSFP imaging was particularly useful for iterative correction, since SSFP produces very high-contrast images with dark bands occurring at fixed frequency increments (∆*f* = 0.66/TR), when employing sequences with set repetition times (TRs) (23). Since this ∆f also reflects the field inhomogeneity, $\Delta f=\left[\gamma \Delta B_{0}/2\pi \right]\text{,}$ where γ is the proton gyromagnetic ratio, it is possible to easily visualize the magnetic field inhomogeneity within a given region. Therefore, increasing the spatial distance between adjacent bands is a rapid indication of the improved homogeneity within this area, so we strove to reduce the number of bands within the desired ROI (such as portions of the LV) while iteratively changing the current magnitude or shim coil location.

## Results

### MRI safety

A set of safety and performance tests were performed combining imaging phantoms and the CSS system prior to its approval for use in animal models. These consisted of B1 measurements, SNR measurements, force balance checks, gradient coil coupling measurements, distortion measurements and heating measurements.

We placed the shim coil alone on the MRI stretcher and validated that the coil did not displace or rotate when we inserted the coil into the bore, as well as when we delivered current into the shim coil. Similarly, we tested the cantilevered system after inserting it into the magnetic field and found that the beam was not displaced by the magnetic field.

Measurements of the reference power and SNR with versus without the CSS system were not significantly different. Gradient coil coupling to the shim coil was evaluated by observing the induced current through the shim coil during an MRI scan. With proper setup of the Techron amplifier the induced current during all MRI sequences studied was negligible.

Although we did not perform explicit heating tests, we did not see signs of additional subject rf heating during MR imaging. The shim coil assembly was operated for several hours at 10A continuous current without significant warming to the touch. SAR values and B1 strength were measured to be similar in phantoms with versus without the CSS system present in the bore tube.

To date, phantom and animal testing has been performed with the ICD boxes with lead wires removed. As the leads themselves do not generate any significant magnetic-field artifacts, ICD’s used with or without leads should present equivalent magnetic field inhomogeneities. Rf heating of the body due to the lead wires is not a major concern at 1.5 Tesla, which is where we conducted our study, as evidenced by the thousands of ICD patients that have safely undergone MRI imaging at this field strength. We discuss high-field rf heating concerns later on in the Conclusions section.

The CSS outer frame was completely constructed of non-conductive materials, insulating a person who touches it from DC or AC electric voltages or currents.

### Phantom testing

#### Measuring image spatial distortion due to CSS

The spatial distortion due to the presence of the CSS system was observed to be primarily along the Y (Anterior–posterior) direction. With the AE Techron 7,224 or 7,234 amplifier, geometric distortion at 0 Ampere current output from the amplifier was reduced to ~5%, as measured by subtracting GRE images in successive acquisitions with changed phase-encoding directions.

#### Correcting B_0_ inhomogeneity interactively in phantoms

Strong artifacts in phantoms were produced by placing ICD cases directly above the phantom. In Figure 5, correction of the artifact is performed by increasing the current and observing with real-time imaging the ensuing changes. Figure 6B illustrates the role of SSFP’s prominent band artifacts in providing a real-time “B_0_ field map,” since the distance between adjacent bands in the field maps indicates the level of inhomogeneity.

### Swine testing

#### Interactive correction of the B_0_ field in swine

**Figure 7:**
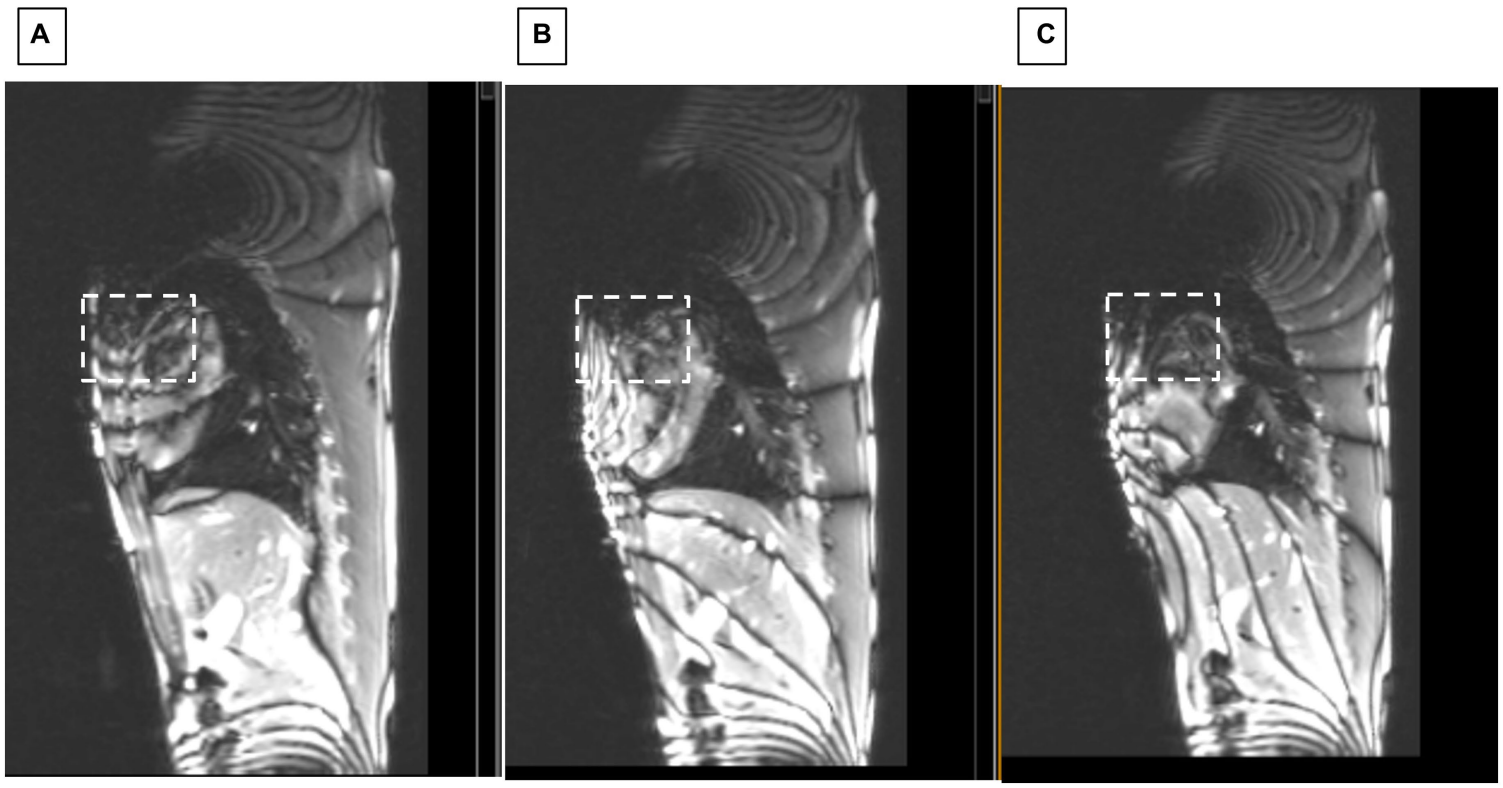
Sagittal SSFP of swine with ICD. Showing changes at applied shim currents of **(A)** 0, **(B)** 2.5, and **(C)** 3.5 Amperes. White dashed square indicates region where homogeneity progressively improved.

After moving the cantilevered shim coil to the B_0_ map estimated location, we performed gross changes in the shim current in order to rapidly estimate the current required. Figure 7 shows how sagittal SSFP images provided a fast approximation of the corrective current required.

#### 2D cine

**Figure 8:**
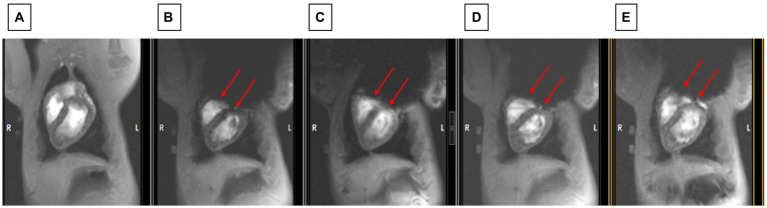
Observing changes during changes in shim coil positioning with cine imaging. **(A)** The state before the ICD was added (which serves as the baseline for restored image quality). **(B)** Worst post-ICD state, **(C)** slightly improved post-ICD state, **(D)** increasingly improved post-ICD state, **(E)** most improved post-ICD state. Note that LV visualization in **E** is almost as good as in **A**. Red arrows point to inferior border of void which is continuously moved between **B** and **E**.

Another sequence that permitted rapidly observing improvement in shimming in the swine was execution of 2D ECG-gated multiphase GRE scans during the repositioning of the shim coil location. Figure 8 shows how improved positioning can provide an LV image similar to the baseline state (with no ICD above the chest), which is shown in Figure 8A.

#### 2D coronal LGE

**Figure 9:**
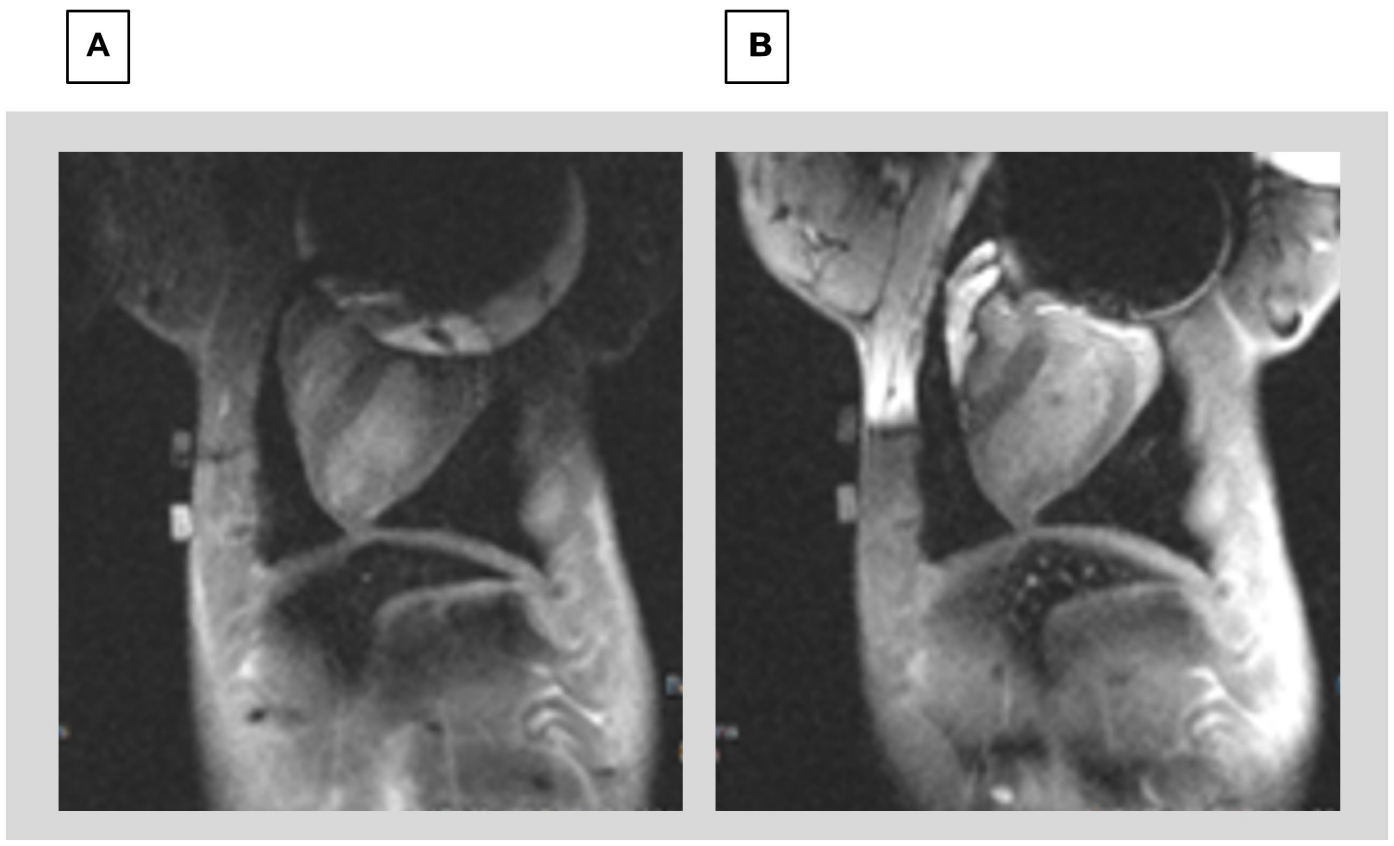
Comparison of 2D LGE **(A)** prior to shimming and **(B)** after shimming using the CSS system. The image prior to shimming has severe distortions at the top of the Left ventricle, whereas the image after shimming allows visualizing the entire LV.

Swine breath-held ECG-gated 2D LGE scans were conducted to validate the ability to correct ICD-related artifacts. Once the shim coil location and current were optimized, it was possible to validate that the shim values were also correct for 2D LGE. Figure 9 compares 2D LGE images acquired before and after shimming.

**Figure 10:**
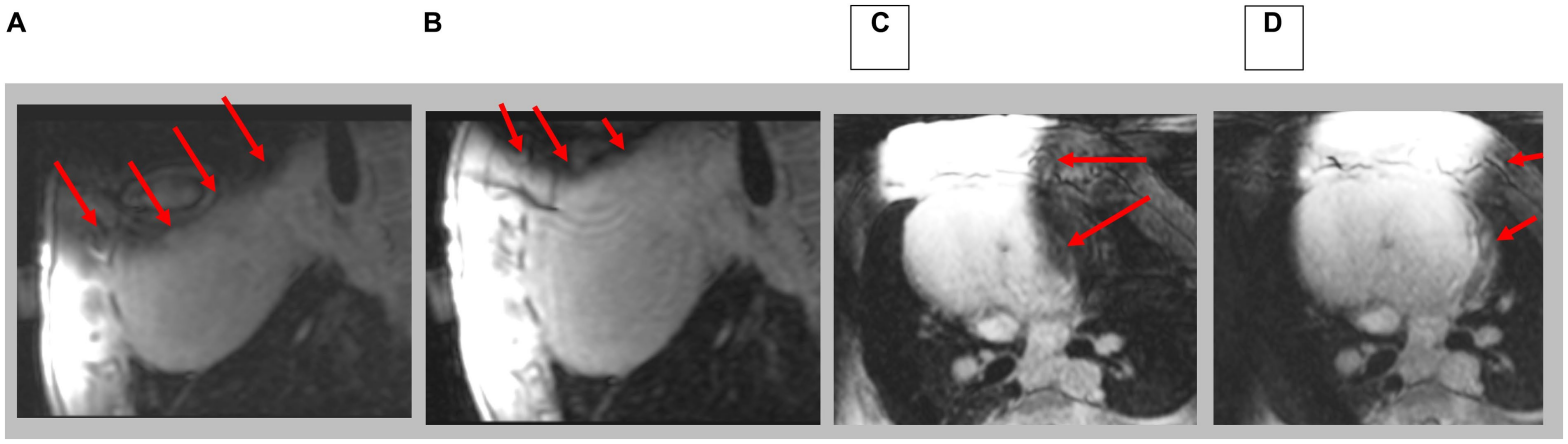
Navigated 3D LGE in sagittal and axial direction; prior to **(A,C)** and after **(B,D)** shim correction. Red arrows indicate location of artifacts. Note complete axial image in **D**.

3D Navigated LGE imaging was performed in the swine using ECG-gating and prospective respiratory navigators. In Figure 10, images can be compared between before and after shimming. Since the scan times for 3D LGE were ~ 6 min, the 3D results demonstrate the temporal stability of the CSS system.

## Conclusion

We have created an external shim coil with a current geometry that corrects some but not all, of the dipole field inhomogeneity created by the ferromagnetic ICD box. It is possible to create a local magnetic field which shifts the local Larmor frequency of a user-defined ROI in such a way that the MR spins become visible within that ROI. This is what the current CSS system performs in a workflow-efficient manner, since it is able to deliver large currents of >720 AmpereTurns without affecting the performance of the MRI scanner, and as a result of the robotically controlled positioning of the shim coil, it can be brought to any desired location within the bore.

As a limitation, It is important to point out that even small ferromagnetic objects can produce very large magnetic field inhomogeneities in their proximity which the CSS system may not be able to correct. We are currently unable to correct inhomogeneities greater than ~100 ppm, although this may be possible with further development.

The current CSS can correct inhomogeneities ~80 ppm but the site of maximum correction may not overly the desired ROI. We are in the process of optimizing coil positioning by a closed-loop system that (1) optimizes fitting of the CSS field to the image distortion field-map in order to minimize the inhomogeneity measuring the image field distortion before CSS, (2) moves the shim coil to the optimized location using robotic positioning control, and (3) repeating this process until the observed inhomogeneity in the RO1 is minimized.

We tested the CSS in phantoms and swine. The CSS is unique in that it provides a far larger shimming capability than possible with currently available shim systems. Additionally, the remotely controlled cantilevered displacement of the CSS system is unique in its ability to bring the shim coil to the exact location where it provides maximal inhomogeneity correction. In addition, the remotely controlled CSS system allows shimming optimization without requiring the MRI technologists to enter the MRI room.

While the CSS system was not tested for its workflow efficiency, we believe that the ability to control all its features from outside the MRI suite will make its use effective in detecting scar resulting from Myocardial Infarction in patients implanted with ICD.

The CSS system is designed to create a local B_0_ “step” which only corrects a strong localized inhomogeneity. If it is desired to obtain an image over a larger ROI with less artifacts, then several images can be collected with different currents and position of the shim coil. These images can then be combined or “stitched” to produce the corrected image. The implementation of this technique is planned for further development.

The CSS system can potentially also be used in patients implanted with other invasive active devices, such as spine and brain stimulators, although validating that will require future work.

The results of this study are currently limited to its use in 1.5 Tesla MRI scanners and at lower field. This is because we do not address possible radio-frequency heating from the ICD lead wires. We know from the multitude of clinical work [thousands of ICD patients already scanned (24)] that imaging in the presence of ICDs can be safely performed at 1.5 T. However, there is increased concern at higher fields (3 T and above) with ICD lead heating, which calls for using sequences with low RF deposition. In future 3 T and higher field implementations of the CSS system, we will thoroughly test for rf heating and develop the means to retire possible patient risk.

## Data availability statement

The datasets presented in this article are not readily available because data is available upon request subject to IACUC restrictions. Requests to access the datasets should be directed to ES, eschmi17@jhu.edu.

## Ethics statement

The studies involving humans were approved by John Hopkins University School of Medicine IRB. The studies were conducted in accordance with the local legislation and institutional requirements. The participants provided their written informed consent to participate in this study. The animal study was approved by John Hopkins University Animal Care and Use Committee. The study was conducted in accordance with the local legislation and institutional requirements.

## Author contributions

All authors listed have made a substantial, direct, and intellectual contribution to the work and approved it for publication.
